# Supplementary figures and images for: A behavioral and modeling study of control algorithms underlying the translational optomotor response in larval zebrafish with implications for neural circuit function
Source: PLoS Comput Biol. 2023 Feb 23;19(2):e1010924. doi: 10.1371/journal.pcbi.1010924 (PMC9998047; doi:10.1371/journal.pcbi.1010924)

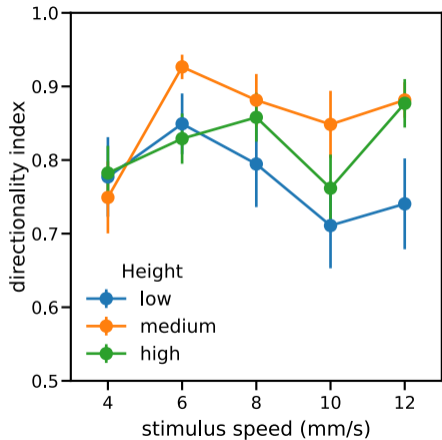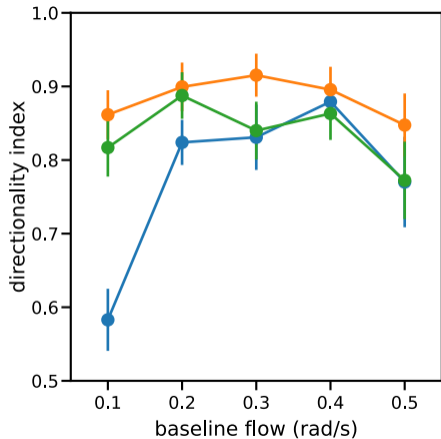

Supplement: S1 Fig — Proportion of swim bouts in the same direction as the stimulus for the OMR regulation experiment (left) and baseline flow experiment (right). (PDF) [file pcbi.1010924.s001.pdf]

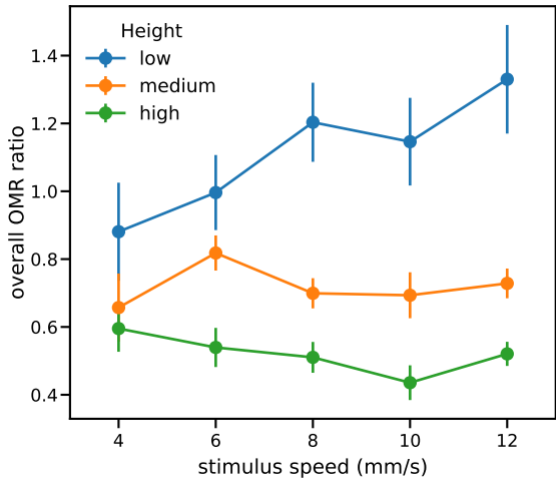

Supplement: S2 Fig — Overall OMR ratios for the OMR regulation experiment calculated over the full trial period up to when the fish first reached the end of the channel rather than during OMR trajectories only. As for OMR performance during OMR trajectories, the OMR ratio decreases systematically with height. As expected, the overall OMR ratios are lower than those observed during OMR trajectories, with both high and medium height groups swimming more slowly on average than the stimulus (OMR ratio less than 1) while the overall OMR ratio for the low height group averaged over all stimulus speeds does not differ significantly from 1. (PDF) [file pcbi.1010924.s002.pdf]

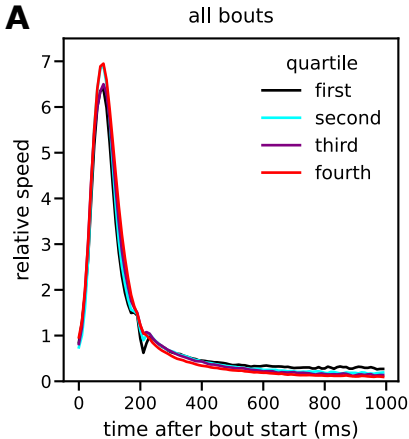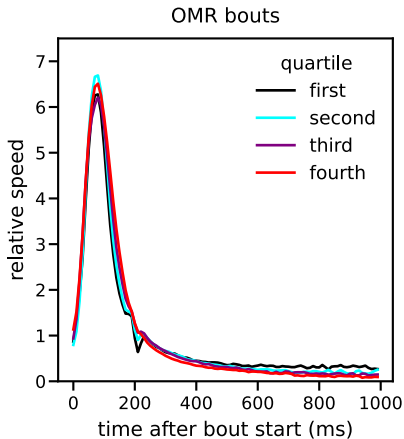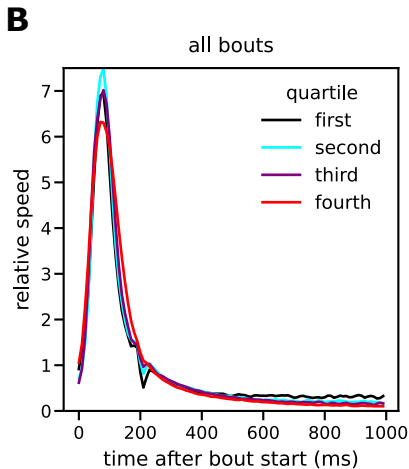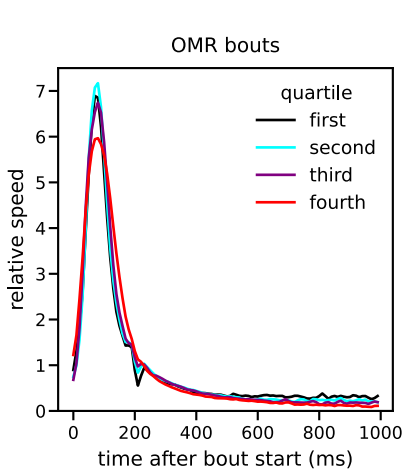

Supplement: S3 Fig — Separate relative speed profiles for bouts split into quartiles by measures of overall bout intensity other than bout initial speed: peak bout speed (A) and total bout displacement (B). The relative speed profiles are again essentially independent of bout intensity whichever measure is used and the profile for OMR-consistent bouts is similar to that for all bouts taken together. (PDF) [file pcbi.1010924.s003.pdf]

OMR regulation experiment

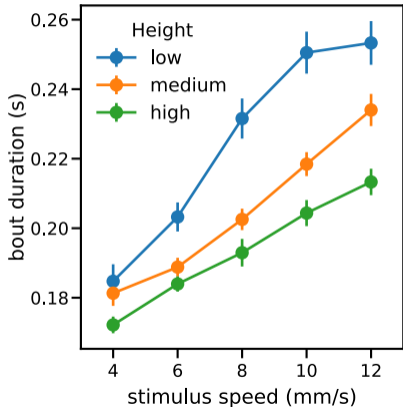

Baseline flow experiment

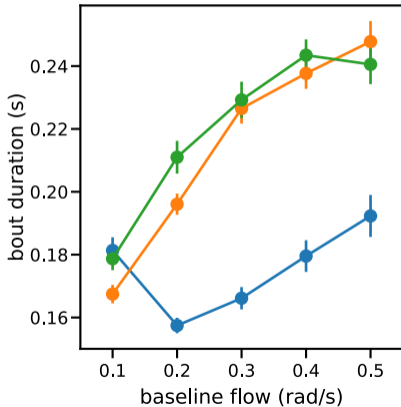

Supplement: S4 Fig — Bout duration for the OMR regulation and baseline flow experiments. The fixed bout speed profile gave rise to bout durations that increased with stimulus speed and baseline flow. Here bout ending was defined as the point during a swim bout when the swim speed, averaged over a 50 ms window, first dropped below 5 mm/s. The OMR regulation results are consistent with, and reinforce, those reported by Severi [25], who found that bout duration estimated from measures of tail curvature increased steadily with stimulus speed for this range of low to moderate speeds. (PDF) [file pcbi.1010924.s004.pdf]

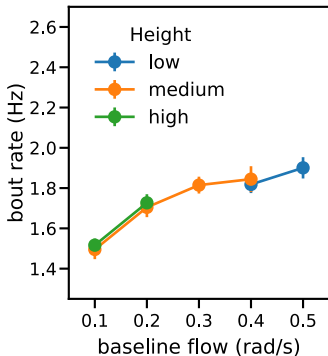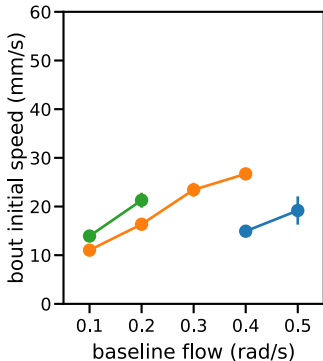

Supplement: S5 Fig — Bout rate and initial speed for the baseline flow experiment, showing only experimental conditions with stimulus speed at least 3 mm/s and at most 15mms to exclude most spontaneous bouts (more likely to occur at slow stimulus speeds when the OMR may not be fully engaged) and fast bouts (mostly occur for speeds faster than this). For a given baseline flow, there is no suggestion that bout rate depends on height. Unlike the OMR regulation procedure, for this procedure the impact of height on mean swim speed is due to variation in bout speed but not bout rate, suggesting that control of bout initiation and bout intensity are decoupled. (PDF) [file pcbi.1010924.s005.pdf]

**A**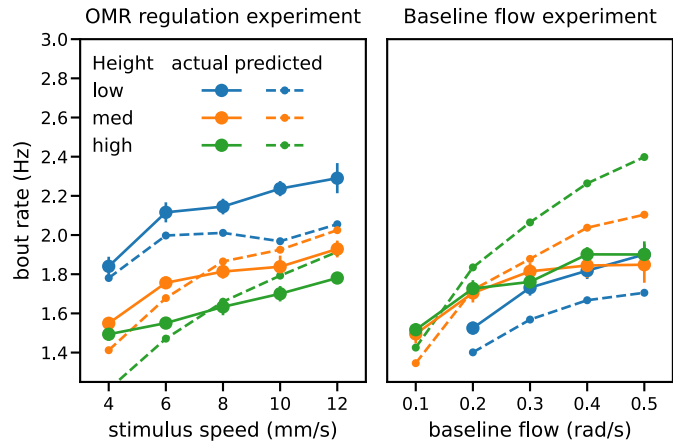**B**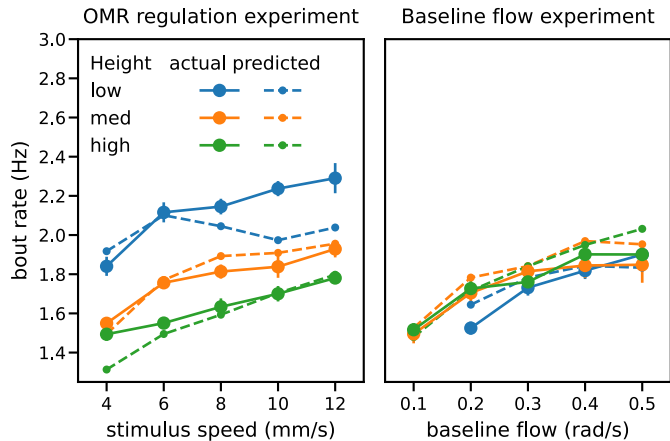

Supplement: S6 Fig — (A-B) Model predictions for bout rates when bout intensity is set to the mean observed values for each experimental condition. (A) Basic bout generator. For the baseline flow procedure, observed bout rates are insensitive to height but the basic model cannot reproduce this finding. (B) Enhanced bout generator with motor inhibition. The qualitative findings can now be reproduced. (PDF) [file pcbi.1010924.s006.pdf]

**A**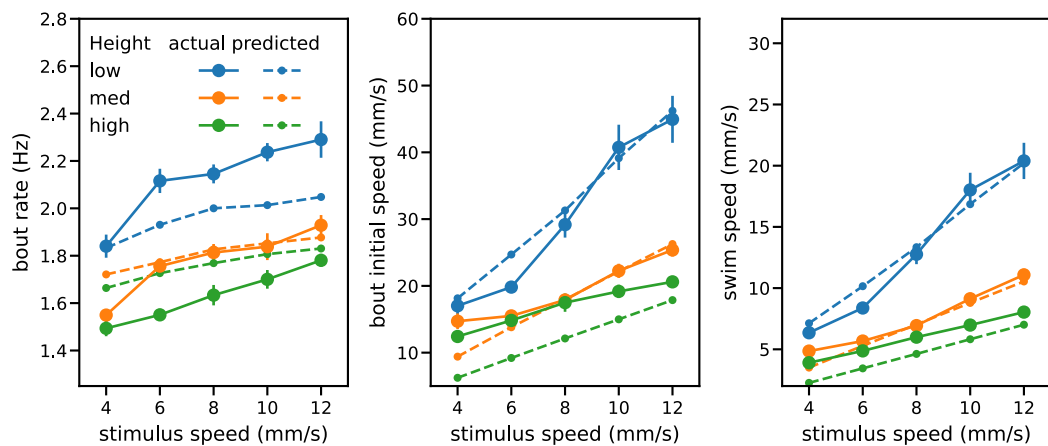**B**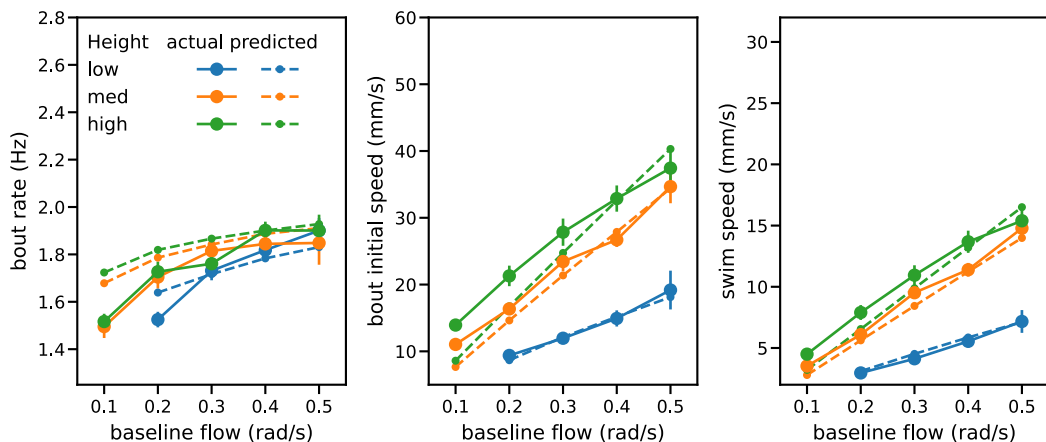**C**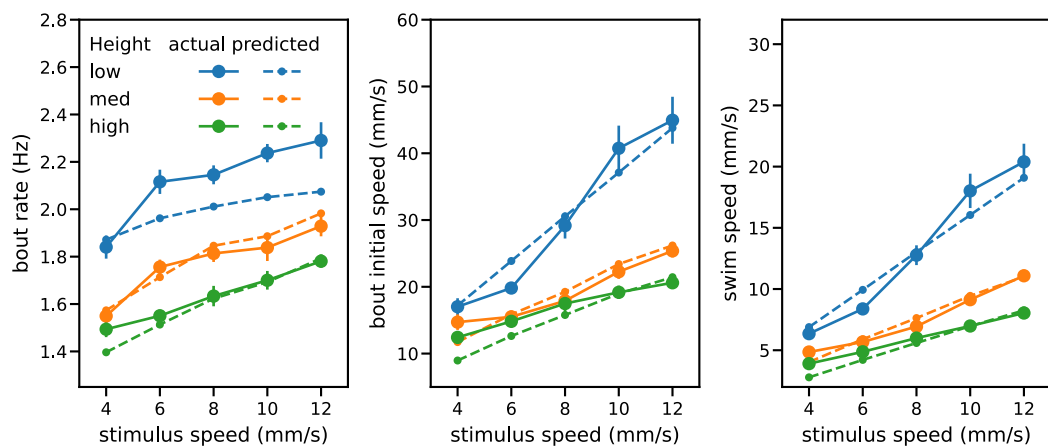**D**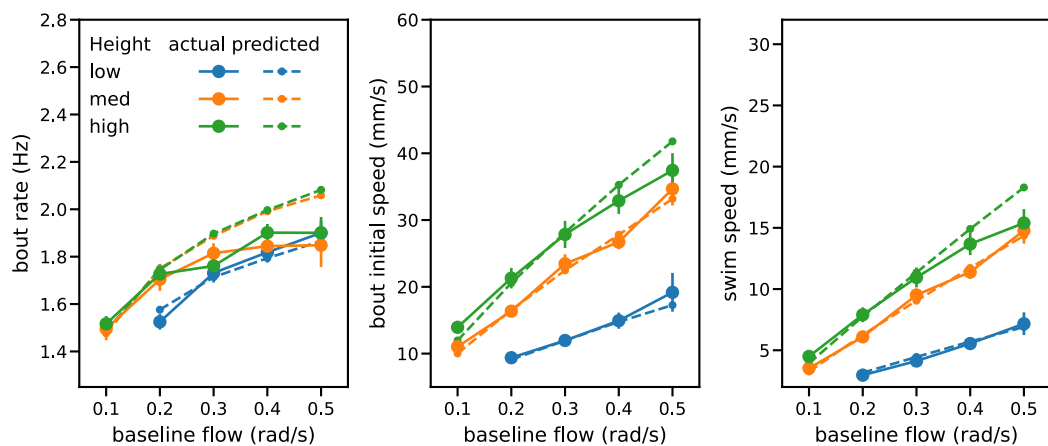

Supplement: S7 Fig — Performance of two-factor model variants A and B. (A-B) Comparison of observed bout rates, bout intensities and swim speeds for the OMR regulation procedure (A) and baseline flow procedure (B) with predictions from model variant A. (C-D) Comparison of observed bout rates, bout intensities and swim speeds for the OMR regulation procedure (C) and baseline flow procedure (D) with predictions from model variant B. (PDF) [file pcbi.1010924.s007.pdf]

**A**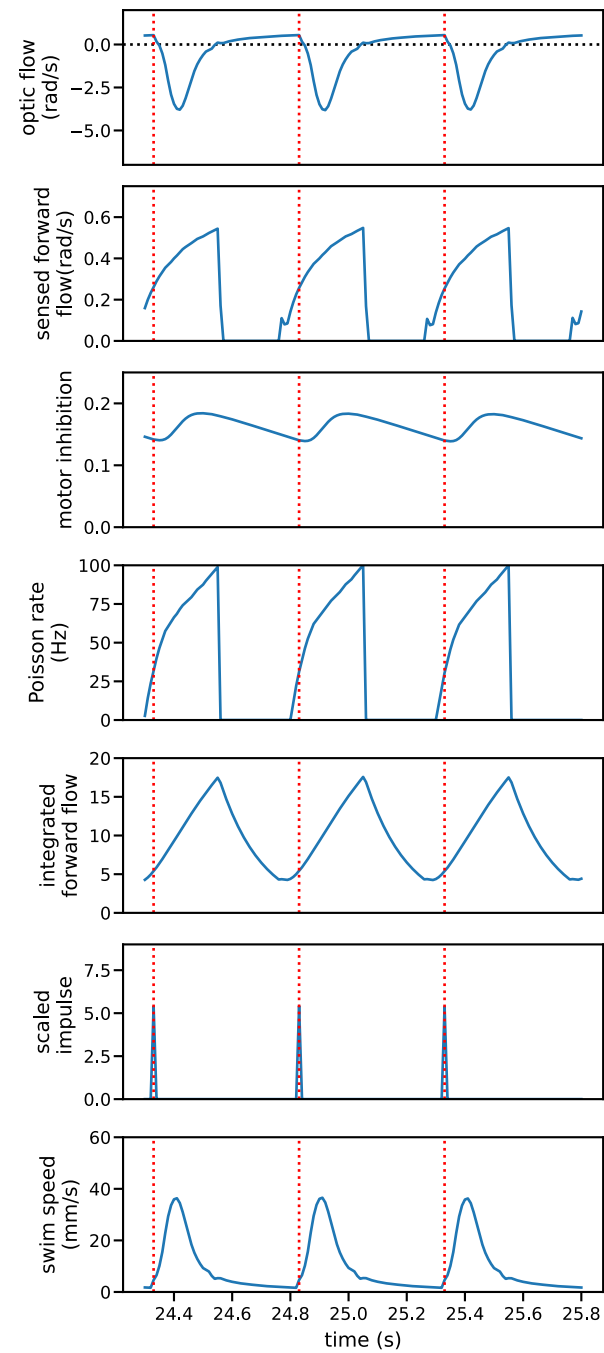**B**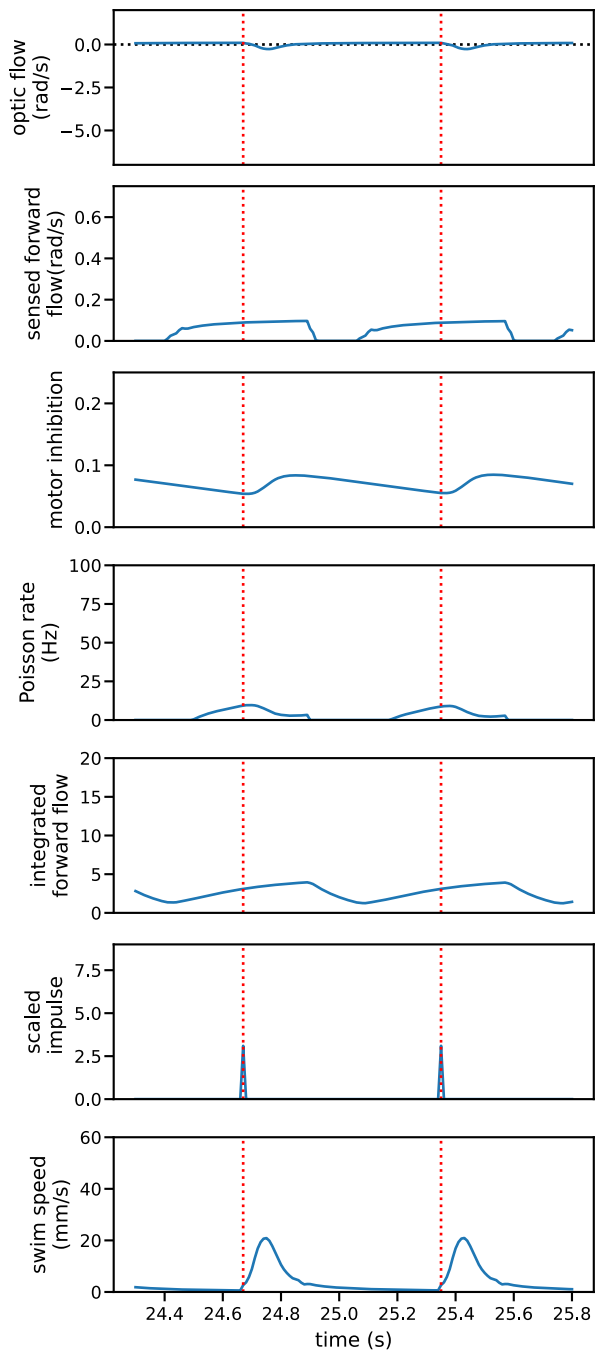

Supplement: S8 Fig — Sample traces for key variables of the final model (dual process model variant C) when stimulus speed is 8 mm/s in the low height (A) and high height (B) condition, corresponding to baseline flows of 1 radian/s and 0.14 radian/s respectively. Red dotted lines indicate the time of bout initiation. Bout rate and bout speed are both greater for the lower height. (PDF) [file pcbi.1010924.s008.pdf]

**A**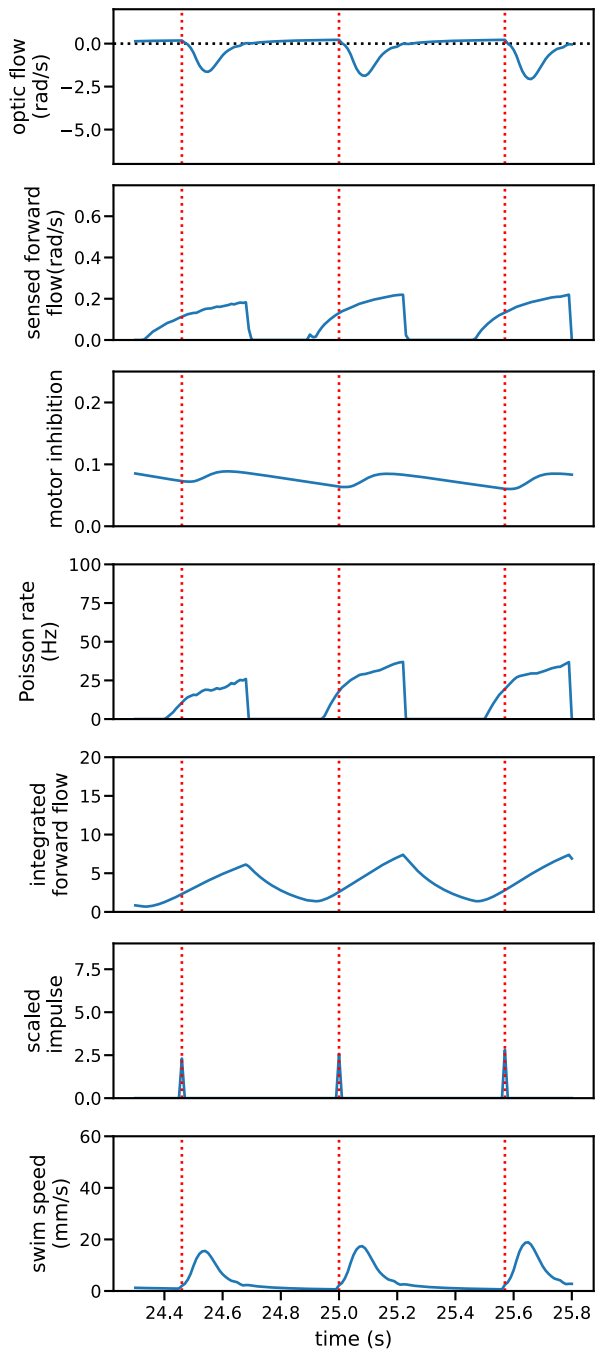**B**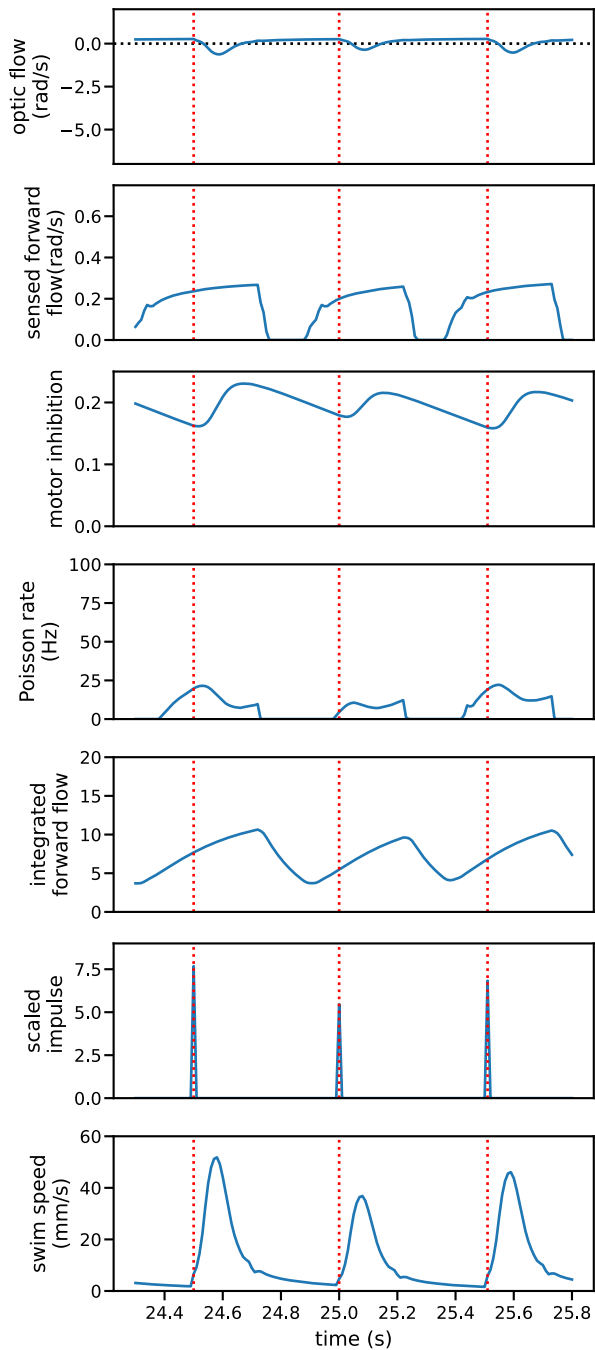

Supplement: S9 Fig — Sample traces for key variables of the final model (dual process model variant C) when baseline flow is 0.3 radians/s in the low height (A) and high height (B) condition, corresponding to stimulus speeds of 2.4 mm/s and 16.8mm/s respectively. Red dotted lines indicate the time of bout initiation. Bout speed is lower for the lower height, but bout rates are about the same. (PDF) [file pcbi.1010924.s009.pdf]

**A**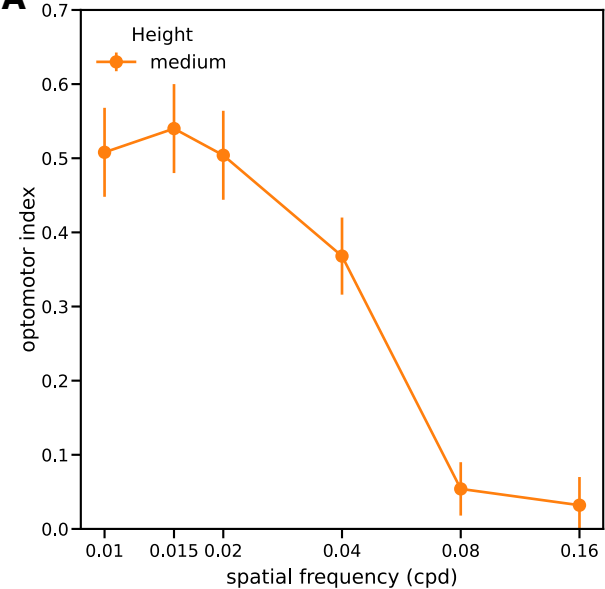**B**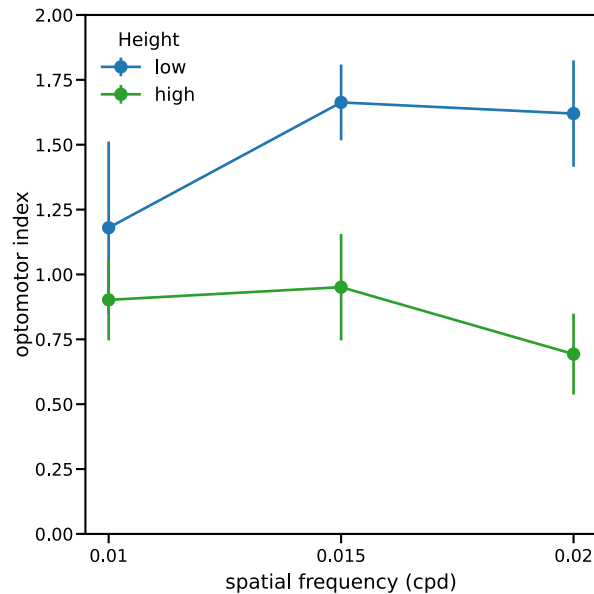

Supplement: S10 Fig — (A) Preliminary experiment to find the spatial frequency (in cycles per degree) of the sinusoidal grid stimulus most effective in eliciting an optomotor response when testing at the medium height (28-36m) with a stimulus speed of 5 mm/s. 24 7dpf AB wildtype zebrafish larvae were tested at each spatial frequency, with the grid moving first forward and then backward for two test traverses each 60s in duration. The optomotor index shown here is the ratio of total distance swum in the direction of the grid stimulus divided by distance moved by the stimulus. It is similar to the OMR ratio measure used elsewhere except that it is based on swimming over the whole trial period rather than only during OMR trajectories. The spatial frequency (0.0156 cpd) used in all subsequent experiments was close to the optimal value of 0.015 found here. The absolute grid period was varied in proportion to height to keep it as constant as possible. (B) A follow up experiment checked that the same spatial frequency is also effective at the low (4-12mm) and high (52-60mm) heights. There were 8 larvae in each experimental condition, with each individual larva experiencing a single height and all spatial frequencies. The frequency of 0.15 cpd was again at least as effective as either 0.01 cpd or 0.02 cpd, suggesting that any variation in perceived grid spatial frequency due to differences in the degree of refraction experienced at different heights did not have a material impact on the strength of the OMR in these experiments. (PDF) [file pcbi.1010924.s010.pdf]

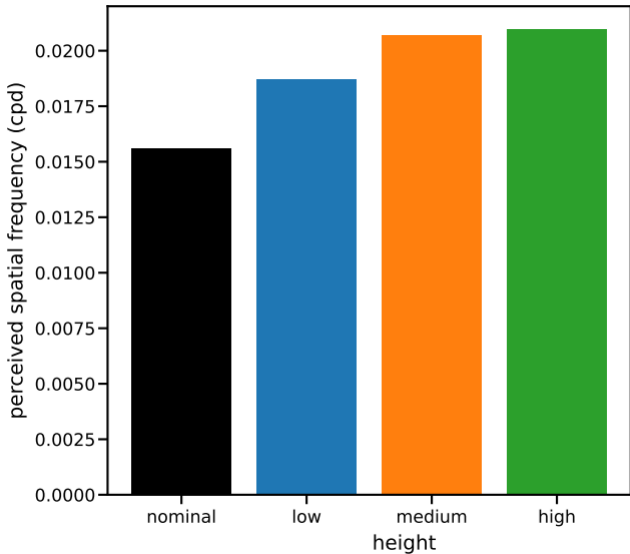

Supplement: S11 Fig — The leftmost bar shows the “nominal” spatial frequency of the grid stimulus that would been perceived in the absence of refraction for all three height conditions. However refractive effects increase the perceived spatial frequency to an extent that depends on the height of the channel above the stimulus (righthand three bars). Following [27] here we treat the bottom of the channel as an air/water interface. The perceived spatial frequency was estimated by calculating the angle subtended at the eye by rays stemming from the limits of a single stimulus period centered directly below. For the values used in these experiments, the three mid-channel heights of 8mm, 32mm and 56mm above the stimulus and a nominal spatial frequency of 0.0156 cycles per degree (cpd), the perceived spatial frequency would have varied from 0.0187 cpd to 0.021 cpd. Given the spatial frequency tuning curve (S10A Fig), this degree of uncontrolled variation (± 7.7% relative to midpoint of the range) about a point close to the peak of the curve would not be expected to have much effect on OMR performance, as confirmed empirically by the subsequent experiment (S10B Fig). As refractive effects increase towards the periphery this may also suggest that the stimulus region directly below the fish has the strongest influence on the strength of the OMR. (PDF) [file pcbi.1010924.s011.pdf]
