# Supplementary material for: A behavioral and modeling study of control algorithms underlying the translational optomotor response in larval zebrafish with implications for neural circuit function
Source: PLoS Comput Biol. 2023 Feb 23;19(2):e1010924. doi: 10.1371/journal.pcbi.1010924 (PMC9998047; doi:10.1371/journal.pcbi.1010924)
Supplement: Attachment — Submitted filename: Review of ZF-OMR Manuscript.docx [file pcbi.1010924.s012.docx]

**Review of ZF-OMR Manuscript**

Hohman et al. PLoS- Computational Biology

**General Impressions**

This manuscript by Hohman et al. examines the OMR of zebrafish revealing new details of this essential vertebrate behavior. By adjusting the optical flow stimulus and tracking swim bouts (with high speed imaging) they observed new elements of OMR neural control. These results are enabled by automated tracking and analysis of batches of larval swim bouts together with a leaky integrator model and discrete time approximation. Of note are methods to capture the initiation and intensity of the individual swim bouts as well as a simulation model with cost function and optimization. Given the impact of zebrafish neural systems, this modeling study brings insights that go beyond the OMR-behavior proper. The optically accessible CNS of the larval zebrafish, whose 100,000 neurons can be imaged on a second by second basis AND optically controlled, provides a wonderful testbed for exploring, manipulating and modeling the neuronal circuits underlying a diverse array of elegant, adaptive behaviors. The Hohman manuscript is a nice addition to this field. I do have questions in regards to interpretation of data and its discussion.

**OVERALL**: A central theme of this work is that the OMR is not fully regulated in that if the vertical position of the fish is raised or lowered above an intermediate level/height (above the striped substrate) the fish either gains or loses ground relative to the apparent motion of the substrate. This result is nicely conveyed and is further addressed in their modeling studies (if I understand the models correctly). I must confess that the model variants and their extended presentation, in which deviations between model and data are incrementally addressed (pp. 9 to 18) are such that I am not exactly sure how to relate the modeling stages/outcomes to their main findings. This includes the mechanisms and significance of the height-effect (as mentioned below). The individual steps are easy to follow, but perhaps some additional in-progress notes might better convey these elements of the manuscript.

**Figure 1** nicely illustrates the optic flow concept and especially how it varies with height above the substrate (e.g. stream bed). I am unclear if the width of the swim channel allows reversal of direction or if the larvae often swim along with the direction of fluid flow? I presume that their automated-tracking ensures that such direction changes and swim bouts would be detected and excluded from analysis of the OMR-driven behaviors.

**Terminology**. Exactly how forward slow-swim bouts and pauses fit into their analytical approach (and their findings) is not exactly clear to me. I can’t say exactly where to insert this essential aspect of larval zebrafish OMR into their methods/results. In particular, I am unsure how their INTENSITY function maps onto the parameters of bout-speed, duration and pauses which (together with heading) should determine the complete trajectory within the swim channels. The introduction (page 3) says that “the relative speed profile of individual swim bouts takes an invariant form that is essentially independent of bout intensity”, while in the Results it says that bout intensity “is the initial bout speed (the mean speed over the first 100ms).” Later on (p. 9) it says that “initial speed, peak speed and bout displacement are essentially equivalent as measures of bout intensity.” In zebrafish swimming, the term bout-intensity is used by Severi-2018, but appears to be equated with maximum tail-beat frequency, which seems very different from the new definition used here. I cannot say exactly what their term “intensity” means, but as used here it seems to conflict with published literature.

**Recordings and Larval Kinematics:** I appreciate the need to live-process the high-speed image stream so as to make recording and analysis manageable, but I have several questions here. I am not suggesting changes to the manuscript, but would just like to better understand the behavioral picture. My first question is whether or not complete high-speed images of channel-swimming been recorded? That is, have images of larvae doing OMR-induced behaviors or other in-stream swimming been recorded so that posture, trunk kinematics, tail-beat frequencies and the like can be analyzed? It’s not clear if this would inform the behavioral analytics or modeling, but being able to compare some exemplar kinematic details with the larger high-throughput dataset might prove useful, at least to relate data-set inferences to ground-truth whole-larva behaviors. In this vein, one could assess if swim-bout kinematics would impact analysis and/or interpretations, especially given that the cited Severi et al. 2018 article reported kinematic/locomotor changes (pauses, speed, tail-beat frequency, gait, bout duration) during OMRs as the speed of whole-field motion increased (this was also linked to activity in nMLF, complementing the Markov-2021cerebellar data).

I share below some broader thoughts on zebrafish navigation, but one aspect of the ideal-OMR vs. observed-locomotion mismatch might stem from diverging visual scene salience. Larvae presumably swim in some proximity to a natural and complex streambed and so the apparent distance-to-streambed with increasing height might mean different things to the larvae, conveying salience beyond an average rate of whole-field visual motion. As such, I am wondering (as a future consideration) whether the same height-dependent results would ensue from a more naturalistic scene?

**Modeling**. I understand that the outcome of their successive models is a good fit between model and the speed-height function (i.e. OMR regulation) and they have defined all of the steps, but I am still missing an intuitive feel for how the parameter-results relate to OMR regulation with changing height.

**Role of** **OMR**: While their data do not negate a role for the OMR in at least contributing to the “stabilizing” of position relative to the ground (including a forward navigational component), their findings do suggest some additional complexity within the locomotor control system which presumably ensues from some ecological, adaptive needs. I do have trouble translating between the modeling terms/verbiage and the facts on the ground (in the stream actually) in terms of real-world salience. The focus of the paper is “exclusively on…translational OMR”, which is fine, but that does not preclude exploratory, postural and other needs from impacting swim bouts and therefore some of the extracted parameters (perhaps) which might further influence their modeling basis/strategy. Possibly the salience and details of their findings can be better related to the natural OMR, a neural control system of immediate relevance to all moving/sighted vertebrates!

Misc. Items:

p. 2 where they say “we unpick the detailed relationship”, perhaps the word “unpack” is intended?

Fig. 1C: I was using the vertical dotted lines to look for alignment between parameters. The first dotted line intersects a point of maximal rate of position change (steepest slope) on an upper graph but a point of zero speed on the lower graph. I don’t understand this.

p. 8 (and Fig. 2) Inter-Bout Interval is summarized for all swim bouts analyzed, by pooling across conditions (I think) but in Severi-2018 it appears that IBI varies with whole-field motion speed (unless I am misreading something). I am unsure how this possible issue might impact their modeling and interpretations, but changing IBI would modify average-rate of speed across the channel, a detail not captured by the intensity parameter.

Stripes. I am unsure if they tested different stripe sizes to see if that affects the height-OMR regulation relationship.

**Other Food for Thought:** The findings of the authors broach a number of interesting topics, which do not particularly need to be addressed in their article, but are provided here mainly for sharing with the authors.

**Backward Flow vs. Baseline**. Backward Flow can be experienced because swimming is too vigorous, i.e. it is overcompensating for any current flow (so to speak) in which case it should be regulated (slowed) if the goal is to maintain position. But in a low flow environment, backward flow is necessary if the larva is exploring terrain to find food patches. In the modeling it seems like this was addressed by nullifying a single parameter, but in a real stream, it seems these would be two very different goals / intentional behaviors with perhaps distinct control circuits.

*Baseline Optic Flow* is the visual change when a fish is not swimming (per the bottom two paragraphs on page 6). This descriptions notes that this effect and gain are confounded with height and also that behavior can be studied in a fish that is not swimming, in the course of using an unchanging environment in which height is changed. I know this is very clear in their minds, but as a confounded reader I suspect others might benefit from a bit more context or explanation here.

**Height Data**, a key discovery of this work, might be considered and expounded upon in a more general context. “Height” is mentioned only three times in the Discussion (all in the first 3 sentences), mainly as a re-statement of the results. Given that the word height appears 76 times in their article, and that the height-effect is their basis for “casting doubt” [p.3] on the idea that the OMR’s main function is to “maintain position”, it would be of value to integrate this height issue into the broader questions about OMR that are addressed in their Discussion.

I am curious if some of the deviation away from “perfect” OMR responses seen in the locomotor bouts data reflects a composite function that incorporates a general valuation of “holding station” [or moving upstream into oncoming flow] together with a quite essential need to control posture (by orienting into a perceived flow). Other ecological needs (detecting prey, favorable hunting grounds) might also influence more general navigational computations that incorporate OMR-related signals. The discussion is written as if the different ecological functions of OMR-driven and related behaviors are exclusive as opposed to being contributory and complementary. “Into-stream” swimming is going to mean different things in different circumstances: in a very slow flow regime, larvae can make upstream progress enabling them to explore favorable locations to find food for example. Moderate flow rates might allow position holding, whereas stronger currents / optical flow might preclude this, raising the question of why expend energy in forward swimming if one is moving backwards (relative to the streambed)? Swimming downstream in such cases would greatly accelerate motion through the environment whereas swimming into the current (yielding slower net drift) might in theory allow for better surveillance of local regions where prey might be found. Competing needs and flexible navigational controls should be considered.

While height influences visual perception of the environment and streambed, rheotaxic signals are also being integrated and so a more integrative theory might help to explain their height data and modeling results. Maintaining posture is also crucial, since a larva hit broadside by a current might lose posture and navigational abilities for some bit of time, possibly with significant adverse effects. The OMR swim-channel design is a necessary step to better understanding of this complex and integrated neural control system. But larval zebrafish have multiple and (at times) competing needs including predator avoidance and prey detecting/tracking, ecological factors that must fit into general swim control. Surveillance for prey, minimization of predation risk and assessment of streambed features might all be influenced by height above the streambed; such considerations were not discussed.

I do not understand how height factors into any of the foregoing considerations nor if the experimental variation of the height parameter (and its modeling) in the OMR-driven swim bouts results in neuronal computations that diverge sharply from more natural sensory processing and sensorimotor integration. An analogous consideration emerges from studies of mammalian visual cortex: natural scenes elicit very different neuronal responses than assorted simple shapes and motions: it is the initial neural responses and processing that will drive further integrative processes.
