# Supplementary material for: A behavioral and modeling study of control algorithms underlying the translational optomotor response in larval zebrafish with implications for neural circuit function
Source: PLoS Comput Biol. 2023 Feb 23;19(2):e1010924. doi: 10.1371/journal.pcbi.1010924 (PMC9998047; doi:10.1371/journal.pcbi.1010924)
Supplement: Attachment — Submitted filename: OMR reviewers.docx [file pcbi.1010924.s013.docx]

Responses to the reviewers

We thank all the reviewers for their thorough and constructive reviews. Their suggestions have led to substantial revisions which we believe have helped us improve the manuscript considerably.

**Reviewer #1**

This study investigates the adequacy of the optomotor response for stabilizing body positions and develops a computational model that accounts for some of the key features of this behavior. The optomotor response (OMR) is a reflex behavior triggered by whole-field visual motion and is found in all vertebrate species. In zebrafish it has recently been investigated in detail and provides an important paradigm for studying sensorimotor processing.
The major finding of the manuscript is that OMR stabilization performance is bad at high and low swim heights, i.e. the swimming velocity does not match stimulus displacement. This suggests that the function of this behavior is not really position “stabilization”, which is a wide-held assumption. Furthermore, the authors characterized the modulation in swimming speed, bout frequency and duration and built quantitative models for explaining and predicting OMR responses to optic flow stimuli varied in distance and speed. This model shows that swim speed “adaptation” can easily be explained by leaky integration of the perceived optic flow, while additional model features (separation of forward from backward optic flow signals, refractory period for inter-swim-bout intervals) are necessary to achieve a good model fit accounting for observed swim bout rates and intensities as well . Such a modeling approach is useful to decipher the control strategies of the behavior and a similar model has already been put forward very recently (Markov et al. 2021).

While the study nicely challenges the assumption that zebrafish achieve stabilization with OMR and provides an interesting -albeit not entirely new- modeling approach towards the OMR control algorithms, there are also a few major caveats that limit the usefulness of the model and possible interpretations regarding the biology. Therefore, the scientific advance the study provides in its present form is limited.

Major concerns:
1) Processing steps of the raw data need to be presented and results need to be embedded in the existing literature. The authors claim that the bout speed profile has a fixed form. However, previous literature showed that bout speed durations are variable (Severi et al. 2014). The authors need to consolidate the different findings and do their best to reveal the origin of this experimental discrepancy. My impression is that the recorded data is of lower spatiotemporal resolution, and the noisiness - together with differences in tested speed regimes between Severi et al.(2014) and this manuscript - might have caused the discrepancy

We have revised the text to make it clear that “bout duration” here as in Severi et al (2014) refers to the duration of active swimming, not the period from one bout to the next (which includes the interbout interval). We also emphasize that it is the *relative* bout speed profile that has a fixed form and explained why this is not in conflict with previous findings that absolute bout duration increases with grid speed (lines 348-354). In fact, because absolute bout speed increases with stimulus speed, a fixed relative profile predicts this observation whenever the end of active swimming is detected by variables such as amplitude of tail or head movements or swim speed falling below some threshold value. This is because absolute swim speeds during the bout are determined by the relative profile scaled up by the overall strength of the bout whether measured by initial bout speed (Fig 2), peak bout speed (A in S2 Fig.) or total bout displacement (B in S2 Fig.), so measurements related to swim speed can be expected to fall below a fixed threshold at a later time for stronger bouts. To illustrate this, we have added S3 Fig. which shows that in our experiments active bout duration increased with stimulus speed in a similar way to that reported by Severi et al (2014). We therefore believe our empirical results are consistent with, and reinforce, those of Severi et al rather than being in conflict (348-354, 1229-1233).

a) Show a raw video of an OMR bout. Show corresponding raw position trace and also the superimposed smoothened position trace. It would be helpful to illustrate the quantitative terms for OMR in this example as well. Now the reader need to search everywhere for the definition of each term.

Fig 2C now shows a sample raw position trace with the portion for a single bout magnified in 2D. It illustrates that at each time step possible positions for the center of mass were identified, one of which was selected in a post-processing step as the most probable true position. Note that no smoothing is involved, as explained in more detail below (c).

Unfortunately we cannot show a corresponding raw video with the spatial resolution that we believe the reviewer is asking for. There was a very good reason for this - the length of the channel in which fish were free to swim was 30 cm. Imaging fish a few mm long at higher spatial resolution would have required moving the camera so that its field of view followed the fish. Although this might have been possible with some sort of motorized gantry system, this would have likely also induced mechanical vibrations sensed through the lateral line system, which also affect swimming behavior, and would have precluded taking observations from several fish at the same time. We therefore decided to stick to the priorities for this study with a high-speed image stream alongside real-time processing while recording position data only.

All quantitative terms are now defined early in the Results section (218-227)

b) Compare speed regimes and also report absolute speeds for experiment 2. Severi et al. 2014 showed there are two modes of OMR bouts, the fast bouts have fixed duration but varied speed while the slow bouts are the opposite. Is the analysed data biased towards modeling the fast bouts only? According to Severi et al., fast bouts are much less frequent than slow ones at a stimulus speed lower than 10mm/s (Severi et al., 2014). Furthermore, the author claimed "...but an invariant relative profile suggests that bout speed and duration share a common causal factor and are not independently controlled..." which is contradicted to the findings in Severi et al. that the speed and duration of fast bouts are independently modulated by stimulus speed. This discrepancy must be addressed before the modeling section.

The stimulus speeds associated with levels of baseline flow at different heights in experiment 2 are now shown in Table 1. Severi et al 2014 found that slow bouts predominated for moderate stimulus speeds, while fast bouts started to emerge at about 12.5 mm/s and increased in proportion thereafter. Here we focused on the control of slow bouts and therefore for experiment 1 employed only moderate stimulus speeds of up to 12 mm/s. Experiment 2 required a wider range of speeds but as discussed below (2b) its conclusions remain unchanged when only moderate speeds up to 15 mm/s are considered. We now make it clear that our conclusions should be taken to apply to slow bouts only (145-149). The general question of a possible discrepancy was discussed above.

c) To what extent do the different smoothing kernels and filters affect the result of the “fixed bout speed profile”? Is it possible that the dominant identified profile is simply caused by the smoothing?

We apologize if we misled the reviewer but smoothing was not applied either during position tracking nor calculation of relative bout speed profiles. The latter were derived from instantaneous swim speeds at different offsets from the bout start time, calculated by taking first-order differences in position without smoothing, by averaging the instantaneous speeds over many bouts and normalizing the result. We have added more detailed descriptions of both these processes to make them clear (920-933 and 963-970). Smoothing was applied as part of identifying the start time of individual bouts but this would not affect the subsequent form of the profiles. In theory smoothing here could have caused the start of strong bouts to be identified earlier than the start of weak ones, in turn causing the profiles for strong bouts to be shifted to the left relative to those for weak bouts. However, as Fig 3F and S2 Fig (A-B) show, there is no suggestion of such an effect: the peaks for the first quartile (weakest) and last quartile (strongest) bouts are closely aligned. The only other application of smoothing was for presentation purposes in what was Fig 1C, as the first-order difference of the position data for individual traces is quite noisy. Smoothing there was the reason why the speed trace appears to be delayed relative to the position trace and why the profiles of individual bouts shown there appear to take a more extended and less peaky form than that of the relative speed profiles. As this trace may have given a false impression, we have removed it in favor of showing the raw position data in more detail in Fig 2C and D.

2) It is unclear to what extent the recorded behavior (at low stimulus speed regimes) corresponds to OMR. The authors had a thresholding approach in which they counted any swims that were fast enough and in the correct direction as OMR. However, even in spontaneously swimming fish, they will sometimes swim quickly into the OMR direction. Figure. 2C (low swim height, blue) shows that at baseline flows of 0.1, 0.2 and 0.3, the swim speed was very low and the data point 0.1 was excluded because it apparently did not contain OMR (but data points 0.2 and 0.3 were included).
a) The authors could quantify an OMR directionality index: what fraction of swim bouts were performed into the “correct” direction in order to judge whether OMR on average occurred or was absent.

Almost all bouts during OMR trajectories were in the same direction as the stimulus (directionality index 0.995 overall) as expected (247). For the trial trajectories as a whole (up to the point where the target was reached) the overall directionality index was 0.826 suggesting that larvae were engaged in OMR behavior most of the time (244-246). With the exception of the excluded data point in the baseline flow experiment, where swimming was much faster than expected for the OMR suggesting the release of spontaneous swimming, this index did not increase materially with increased stimulus speed or baseline flow (new S1 Fig). This suggests that the behavior was consistent with the OMR as much in the low stimulus speed conditions as the high ones.

b) The authors should compare the absolute speed regimes (mm/s) between experiment 1 and 2. To what extent can the different stimulus regimes explain the observed behavioral differences? Is it possible that the “decoupling” of bout rate and bout initial speed (Fig. 2D) is simply due to very low stimulus speeds for the low height in experiment 2 (spontaneous swimming), whereas such low stimulus speeds (and thus the “decoupling”) were simply not used in experiment 1?

The range of speeds required in experiment 2 is indeed much wider, from 0.8 mm/s to 28.0 mm/s, than those used in experiment 1 (new Table 1). However, when only conditions with moderate stimulus speeds similar to those in experiment 1 are included, height still impacted bout speed but not bout rate (359-362, new S4 Fig). The decoupling is therefore unlikely to be due to differences in the stimulus speeds used.

3) The model description is not detailed enough and scattered throughout the manuscript. Which parameters (and what number of them) were fitted and which ones were fixed? What are the resulting parameter values that were used in the model (e.g. the different tau_f)? This should be shown in a table. For example, it was not entirely clear to me, whether the ki (intensity scale factor) was chosen independently for each swimming height (which would make the result of a perfect model fit to swim speed rather boring) or whether the same ki was taken for all swimming heights (the good model fit would be interesting in this case, as it would suggest that there is a single/linear motor “adaption” rule that can explain different swim speeds for different swim heights in experiment 2).

The initial model is now explained in more detail (383-445, new Fig 4B) and the final one summarized at the beginning of the Discussion (715-738), with a complete formal description in the Methods as before (977-1065). Model parameters from fitting are now shown in new Tables 2-4. For a given model all parameter values were the same for all conditions (combinations of height and stimulus speeds) and across both experiments. We agree it would have been trivial to achieve a good fit had they been allowed to vary with height etc.

4) The stimulus setup has a gap of air between display and water body. This leads to refraction artifacts that affect spatial frequency and perceived stimulus velocity (see Dunn and Fitzgerald, Elife 2020, parameter da/dw in Figure 1). This means that the experiment in Fig. 2 is potentially ill-conceived, because the claim of “same flow across heights” is not (completely) true. The authors need to calculate the distortion and replot the data. If the discrepancy of baseline flow (and spatial frequency) is less than 10-15%, then it is probably acceptable. If the discrepancy is larger, the results need to be reinterpreted and potentially experiments repeated.

It is not possible to give a single overall figure for the impact of refraction on spatial frequency as the degree of refraction depends upon the angle of view, being zero for the image region immediately below the fish and increasing towards the periphery. However, to test directly the impact of variations in this factor due to height we have run an additional experiment that suggests the frequency found initially to be most effective at the intermediate height and used subsequently was also optimal at both low and high heights. The impact of height on refractive effects may have been limited here because the fish were mostly responding to optic flow from the region of the ground immediately below the fish, where refraction is minimal at all heights, as they do when responding to local edges (Kist & Portugues 2019). Now addressed in the Results (193-195) with details in the Methods (903-910, 1262-1274, new S9 Fig)

Minor points:
5) Can fish perceive depth? The key assumption for the major point in this study: self-stabilization is not the primary function of OMR, is that fish have access to depth information and do not use it well for adjusting OMR speed. It seems likely that fish do not perceive depth and the differences seen in experiment 2 across swim heights are simply due to the swim-induced optic flow and the integrator according to the model (and possibly partially due to stimulus artifacts). It would help if the authors clearly stated their interpretation of the data regarding depth perception.

We do not assume that the fish can perceive depth. On the contrary, we suggest that the observed effects of depth on behavior can be explained purely in terms of its impact on optic flow, which the fish can perceive, without any need for direct access to depth information. This is now clarified in lines (96-98).

6) The behavioral characterization part has terminology problems which make it difficult to understand and evaluate some results in Figure 1 and 2, some are listed below:
a) How is the term "bout initiation" defined in the section: "Bout initiation and bout intensity are separately controlled"? From what I understood, the bout initiation seems to be an event. Is it quantified as bout rate? Then it would be much clearer to replace "bout initiation" with "bout rate"

Bout initiation refers to the event of a swim bout starting, while bout rate is a summary statistic that captures one aspect of the pattern of several such events. It seems to us this difference in meaning is sufficient to justify retaining both terms.

b) How is mean swimming speed calculated, the total moving distance during the whole 30-second stimulus phases, or is it only calculated for the OMR trajectory?

Mean swimming speed is calculated over OMR trajectories only. This is now clarified at the beginning of the Results (218-220) with further details in the Methods (938-957).

c) How is relative speed defined? What's its unit? And how long is the time window?

Now defined more clearly in Results (327-329) with details in a new Methods section “relative bout speed profile” (963-970). The relative speed at a given time offset from bout initiation is defined as the ratio of the speed at that time divided by the mean speed over the first second following bout initiation. It is a dimensionless quantity.

d) In the definition of "OMR-consistent bout" in the result section, it says in the bracket "see materials and methods section for details", but I didn't find the corresponding method section.

Now defined in the Results (217-218).

7) It is nice to see the model perform (fit) well. But what mechanism in the model actually makes it work? It would be helpful if the authors could plot the perceived optic flow speed (over time) in the model across different heights and relate it to the leaky-integrator model (length of the tau_f time constant) to understand why and at what time points relative to bout initiation the OMR swimming overshoots or undershoots and how the swim vigor gets reduced (“motor adaptation” ) when feedback is large at low swim heights. If the authors illustrated this better, then it would become more clear, how this model does not require “motor adaptation”, but that the observed swim gain adaptation across heights is simply the result of a static leaky integrator that gets charged less at low swim heights due to the large effect of each swim bout on optic flow. Also the results could be discussed in the context of Bahl et al. Nat Neurosci 2020.

We now summarize the overall operation of the final model in the Discussion (715-738), while plots of perceived optic flow and other key variables in the final model have been added to illustrate performance at low and high heights and for both procedures (S7 Fig, S8 Fig). To illustrate how the OMR, though not requiring “motor adaptation”, nonetheless results from a fairly complex interaction of several mechanisms we now also explain how one of our empirical observations – the independence of bout rate and height in the baseline flow procedure – may result from two opposing effects that tend to cancel each other out (696-705).

8) The bout rate is calculated as the frequency of bout during an OMR trajectory which require the fish swim faster than 0.5 mm/s on average. But it is possible that zebrafish larvae, due to their intermittent swimming pattern, solved the over-compensation problem with long inter-bout pause which is left out by this criterion?

We apologize but suspect we don’t fully understand this question. Long inter-bout intervals are indeed a major factor in slow mean swim speeds and would reduce over-compensation, but are included in the model and explained as the result of a low Poisson rate for bout initiation.

9) Please fix the aspect problem in Figure 2G-H and what's the p-value of Pearson's R

We have redone the analysis and redrawn the figures (now Fig 3G-H). The p-value is <0.001 (611-614) in both cases.

10) Please refer to the figures in the discussion section, now it is really hard to find the corresponding results.

These have been added.

11) Fig. 1D: Swim heights should be indicated in the main text. Color usage in panel D and F do not correspond, please consider a use of color that is easier to follow throughout the manuscript.

Swim heights are now included in the main text (207). Colors for bout speed, bout rate and swim speed in prediction error bar charts and for bout categories (quartiles and all vs OMR-only) in profile plots are now distinct from each other and from the colors used for height levels.

12) Line numbers are missing on the manuscript pages. Including them would make it easier to provide specific comments.

We apologize for not including line numbers – these are now present.

**Reviewer #2**

This manuscript is of high interest to anyone using the optomotor response to elicit locomotion or neural activity in zebrafish, but is also more broadly applicable to other systems. The authors begin by investigating something that really needs deeper investigation- namely trying to understand mechanistically what is occurring during the OMR rather than simply using it as a tool to elicit a response, and to understand its potential function for the animal. The introduction is well written and well referenced. I think this is an innovative and significant endeavor, however I was confused at the outset about the gain control, which I attempt to explain below. I believe a revision to make this clearer to your readers would be valuable.

We have added new text (111-127) and added a new figure, Fig 1, in the Introduction which we hope will clarify the concept of feedback gain.

To summarize here, in free-swimming experiments a change in swim speed causes a change in the optic flow feedback signal, where optic flow is defined here as the speed that the image of the grating below moves across the retina. We define “feedback gain” as the ratio of the change in optic flow to the change in swim speed.

For a fish swimming with speed v through the water at a height h above a grid moving at speed s, the optic flow is

$$w=(s-v)/h$$

If the swim speed changes from v1 to v2 while s and h remain constant the resulting change in optic flow is

$$w2-w1=\frac{s-v2}{h}-\frac{s-v1}{h}=-\frac{1}{h}(v2-v1)$$

and the feedback gain is

$$\frac{w2-w1}{v2-v1}= -1/h$$

Feedback gain in this setting is therefore determined by height alone and is inversely proportional to it – it is just the negative reciprocal of height.

(The explanation given in the caption for the new Fig 1 gives a slightly more general derivation, but this is the essence of it).

In fact, we use “feedback gain” here mostly to help relate free-swimming experiments to fixed-head “virtual reality” experiments, as this is a term used in the VR literature. In itself it isn’t that important for understanding most of the material here. So, we generally place less emphasis on feedback gain in this revision and focus more explicitly on the impact of height.

I will just come out and say, because it will be obvious anyway from my review, that I am not a modeler nor an engineer, but a biologist who uses the OMR. Thus, I am highly interested in this manuscript and highly motivated to understand it. I suggest revisions because there were several key items, upfront, that I was not able to understand, simply in the first 2 figures on which I will focus.

Results/Figures 1 and 2 comments:

I think Figure 1 panels A and B are quite clear, important, and nicely done. What is not clear to me leaving panel B is where does the gain measurement occur? Here we are talking about the angle, the optic flow, the height, and the distance, but it would be helpful to discuss gain in this schematic because I do not understand where this is coming from.

The new Fig 1 includes what was previously Fig 1B plus two new panels that we hope will explain this more clearly. As described in the response to your point above, in the free-swimming situation “feedback gain” in this situation is simply the negative reciprocal of height -1/h and so is determined by the choice of height rather than requiring separate measurement.

Panel C is very nice and important to show. What are the dotted lines? This is not explained in the legend.

Sorry if this caused confusion. The dotted lines just gave a subjective indication of where a bout might start and end, and have now been removed as one of the changes to Fig 1C (which is now Fig 2C and D).

It is stated in Panel D that gain decreases as height increases, but I don’t understand why that is the case. I just don’t understand what you’re referring to as the gain here. This is really a main issue for me in understanding the paper. It would be very helpful to explain this explicitly for the less mathematically-savvy reader like myself. I understand how changing the height changes the optic flow. Does changing the height not also change 2 other things: the perceived speed of the grating to the fish, and the perceived period of the grating by the fish? As I understand it, you are manipulating height but could, in fact, achieve the same effect by calculating what the speed and period should look like to the fish at that height and adjusting those parameters. This is all in open-loop, correct? So if it’s not a closed-loop feedback gain you’re changing, I’m still confused as to where the gain comes from. Critically, we know changing speed and period of the grating at a fixed height drastically impacts the response of the fish. In Figure 2 you are compensating for the speed as you change height, but not compensating for the grating period, and claim this keeps optic flow constant, but I don’t understand why as these two visual stimuli are not identical. Would this not be equivalent to putting a fish at fixed height and changing grating period and keeping speed constant? As the fish moves further from the stripes, with a constant width of the screen, they will appear thinner. As someone who has done that experiment for fun, I can tell you changing the stripe width with constant speed and height has dramatic impacts on the behavioral response. Is this what you mean by gain? I’m genuinely confused here and I’d love it for you to spell it out for me very clearly. There is no mention of “gain” in the results text for Figure 1 other than to point out that it changes in panel 1.D. In the results text for Figure 2 you state that “feedback gain and baseline optic flow are both inversely proportional to height”, and say you’re changing the gain, but don’t say much else about it.

Thanks for highlighting this. Changing the height of a fixed grating would indeed change its perceived spatial period as you suggest, with potential impact on the OMR, so in both experiments we increased the spatial period in proportion to height to keep the spatial period of the image on the retina as constant as possible. The relevant panels (now Figs 2B and 3A) were misleading in this respect and have now been changed to make this clear. It is also is now stated at the beginning of the Results (191-195) with details in the Methods (900-910) and we have added S9 Fig A and B to address the spatial frequency issues.

We see the OMR as a closed-loop situation because the fish by swimming faster or slower changes the visual feedback it receives i.e. the optic flow. This is now covered in the Introduction (122-127; new Fig 1C).

Panels E and F. Is low, medium, and high here referring to optic flow, to gain, or to height? This is not clear or stated in the legend. What is “bout initial speed” and how is this measured? I could not find this in the legend or methods. You explain OMR ratio very clearly and well in the results, but is not in the methods or legend and possible should be.

We’ve added a title to the figure legends to make it clear that low, medium and high refer to height. Bout initial speed and the other quantitative measures are now defined early in the Results section (218-227).

Regarding OMR ratio- it’s not clear to me what the problem is for the fish to overshoot this ratio. Why should the goal be to be exactly at 1? It is obvious to see why undershooting it could be problematic, resulting in a failure to compensate for being washed downstream, but if water is being sucked quickly downstream, what is the ethological harm of staying ahead of it? Frankly I would expect that the fish can keep up or overshoot fairly well, especially at these very slow speeds in this experiment, and I don’t see this as a failure. Also, in Figure 1, given that you’re changing the height, the absolute stimulus speed is not the perceived speed by the fish here. Some more clarity on why ratios >1 are problematic from your point of view would be helpful.

We didn’t mean to suggest that “over-compensation” is necessarily a problem for the fish – rather for the common assumption that the OMR results from a regulatory mechanism that holds position over the ground. The Discussion (849-851) now acknowledges that swimming faster than the stream might indeed be beneficial in some circumstances.

Methods comments:

Thank you for picking up on the various omissions here.

1. Which WT line was utilized? Differing WT lines can respond differently to OMR.

AB WT line – added to Methods (887)

2. Is the acrylic arena clear? Can the fish see each other? Could this contribute to their visual responses?

There is a barrier between channels to prevent the fish seeing each other, which might indeed affect their responses. Added to Methods (894-895)

3. What is the actual trial duration for the trial traverses? Is it also 30 seconds like the “initial traverse”?

60s for both traverses. Added to Methods (911-914). Also corrected duration of initial traverse.

4. The width of the channels is not specified.

Width was 10mm. Added to Methods (893)

5. Most importantly, the point about grating speed being kept low. This could impact your conclusions as slower and faster swimming kinematics and bout dynamics vary significantly. While this absolutely should be in the methods, it’s important to point out that this is really a sub-section of the possible OMR responses being explored, rather than the full range of speeds, and thus, the full range of bout dynamics.

Thank you for highlighting this important point. In the OMR regulation experiment we deliberately restricted grating speeds to those which Severi et al 2014 found to elicit mostly slow bouts and our conclusions generally relate only to bouts of that kind. This is now stated explicitly in the Introduction (144-149). In the baseline flow experiment much higher grating speeds were required when swimming was at high level yet the model still fitted those conditions well, suggesting that it might in fact apply also for fast bouts, but no strong claims can be made about that.

**Reviewer #3**

Review of ZF-OMR Manuscript

Hohman et al. PLoS- Computational Biology

General Impressions

This manuscript by Hohman et al. examines the OMR of zebrafish revealing new details of this essential vertebrate behavior. By adjusting the optical flow stimulus and tracking swim bouts (with high speed imaging) they observed new elements of OMR neural control. These results are enabled by automated tracking and analysis of batches of larval swim bouts together with a leaky integrator model and discrete time approximation. Of note are methods to capture the initiation and intensity of the individual swim bouts as well as a simulation model with cost function and optimization. Given the impact of zebrafish neural systems, this modeling study brings insights that go beyond the OMR-behavior proper. The optically accessible CNS of the larval zebrafish, whose 100,000 neurons can be imaged on a second by second basis AND optically controlled, provides a wonderful testbed for exploring, manipulating and modeling the neuronal circuits underlying a diverse array of elegant, adaptive behaviors. The Hohman manuscript is a nice addition to this field. I do have questions in regards to interpretation of data and its discussion.

OVERALL: A central theme of this work is that the OMR is not fully regulated in that if the vertical position of the fish is raised or lowered above an intermediate level/height (above the striped substrate) the fish either gains or loses ground relative to the apparent motion of the substrate. This result is nicely conveyed and is further addressed in their modeling studies (if I understand the models correctly). I must confess that the model variants and their extended presentation, in which deviations between model and data are incrementally addressed (pp. 9 to 18) are such that I am not exactly sure how to relate the modeling stages/outcomes to their main findings. This includes the mechanisms and significance of the height-effect (as mentioned below). The individual steps are easy to follow, but perhaps some additional in-progress notes might better convey these elements of the manuscript.

Operation of the initial model is now explained in a new section and figure (383-445, Fig 4B) and the final one summarized as part of the Conclusions (715-740), including new figures to illustrate how the final model behaves for different heights (S7 Fig, S8 Fig). We hope this will make it easier to understand how the proposed mechanisms generate the observed relationships between environmental conditions (height and stimulus speed) and behavioral outcomes.

Figure 1 nicely illustrates the optic flow concept and especially how it varies with height above the substrate (e.g. stream bed). I am unclear if the width of the swim channel allows reversal of direction or if the larvae often swim along with the direction of fluid flow? I presume that their automated-tracking ensures that such direction changes and swim bouts would be detected and excluded from analysis of the OMR-driven behaviors.

Yes, that’s right. The width was 10mm (now given in the Methods 893) sufficient for the fish to turn and swim against grid movement. Overall, 83% of bouts were in the same direction as the moving grid (244); a new figure S1 Fig shows the overall proportion of such bouts for the different experimental conditions. As you suggest, for OMR analysis we extracted “OMR trajectories” i.e., periods during which the fish was swimming in the same direction as the stimulus (Results 214-218; Methods 934-957). 99.6% of the bouts in these OMR trajectories were in the same direction as the stimulus (247).

Terminology. Exactly how forward slow-swim bouts and pauses fit into their analytical approach (and their findings) is not exactly clear to me. I can’t say exactly where to insert this essential aspect of larval zebrafish OMR into their methods/results. In particular, I am unsure how their INTENSITY function maps onto the parameters of bout-speed, duration and pauses which (together with heading) should determine the complete trajectory within the swim channels. The introduction (page 3) says that “the relative speed profile of individual swim bouts takes an invariant form that is essentially independent of bout intensity”, while in the Results it says that bout intensity “is the initial bout speed (the mean speed over the first 100ms).” Later on (p. 9) it says that “initial speed, peak speed and bout displacement are essentially equivalent as measures of bout intensity.” In zebrafish swimming, the term bout-intensity is used by Severi-2018, but appears to be equated with maximum tail-beat frequency, which seems very different from the new definition used here. I cannot say exactly what their term “intensity” means, but as used here it seems to conflict with published literature.

These issues are now addressed in more detail early in the Results (222-227). The “intensity”, “vigor” or “effort” (all terms found in the literature) of an individual swim bout is rather a vague concept that could be identified with several measurable variables including speed-related variables such as initial bout speed, peak speed or displacement, variables summarizing behavioral details such as tail beat frequency, rostral bend amplitude or head yaw as in Severi et al (2014) or motor nerve firing rate as in some VR studies. We used a swim speed related measure here because swim speed has a direct, exact and easily calculable effect on the degree of regulation achieved by the OMR, our main focus. Other measures will be correlated to some degree with swim speed but do not have that advantage. We chose initial bout speed in particular because its relative bout speed profile proved very similar to the other speed related measures of peak bout speed and bout displacement, suggesting that these are essentially equivalent measures of bout speed, while initial bout speed is less noisy than peak speed and unlike displacement not confounded with the start time of the following bout. However, as you say measures other than bout speed have been identified with “intensity” in the literature as you say so we now generally avoid using that term in favor of “bout speed” to avoid confusion.

Recordings and Larval Kinematics: I appreciate the need to live-process the high-speed image stream so as to make recording and analysis manageable, but I have several questions here. I am not suggesting changes to the manuscript, but would just like to better understand the behavioral picture. My first question is whether or not complete high-speed images of channel-swimming been recorded? That is, have images of larvae doing OMR-induced behaviors or other in-stream swimming been recorded so that posture, trunk kinematics, tail-beat frequencies and the like can be analyzed? It’s not clear if this would inform the behavioral analytics or modeling, but being able to compare some exemplar kinematic details with the larger high-throughput dataset might prove useful, at least to relate data-set inferences to ground-truth whole-larva behaviors. In this vein, one could assess if swim-bout kinematics would impact analysis and/or interpretations, especially given that the cited Severi et al. 2018 article reported kinematic/locomotor changes (pauses, speed, tail-beat frequency, gait, bout duration) during OMRs as the speed of whole-field motion increased (this was also linked to activity in nMLF, complementing the Markov-2021cerebellar data).

We did consider measuring other behavioral analytics but a major technical difficulty was the length of the channel in which fish were free to swim – 30 cm. Imaging fish a few mm long at sufficient spatial resolution to quantify variables such as trunk kinematics and tail beat frequency would have required moving the camera so that its field of view followed the fish. This might have been possible with some sort of motorized gantry system, but this would have likely also induced mechanical vibrations sensed through the lateral line system, which also affect swimming behavior, and would have precluded taking observations from several fish at the same time. We therefore decided to stick to the priorities for this study with a high-speed image stream alongside real-time processing while recording position data only.

I share below some broader thoughts on zebrafish navigation, but one aspect of the ideal-OMR vs. observed-locomotion mismatch might stem from diverging visual scene salience. Larvae presumably swim in some proximity to a natural and complex streambed and so the apparent distance-to-streambed with increasing height might mean different things to the larvae, conveying salience beyond an average rate of whole-field visual motion. As such, I am wondering (as a future consideration) whether the same height-dependent results would ensue from a more naturalistic scene?

This paper focusses on exploring relationships between optic flow and the OMR, and so we used a sinusoidal stimulus whose appearance was as independent of height as possible to minimize other visual cues (81-88). We agree that in the natural setting other visual properties such as perceived texture of the stream bed or the salience of prominent local features will vary with height and this may also impact the OMR. This is now addressed in the Discussion (820-823, 853-536)

Modeling. I understand that the outcome of their successive models is a good fit between model and the speed-height function (i.e. OMR regulation) and they have defined all of the steps, but I am still missing an intuitive feel for how the parameter-results relate to OMR regulation with changing height.

Operation of the initial model is now explained in a new section and figure (383-445, Fig 4B) and the final one summarized as part of the Conclusions (715-740), including new figures to illustrate how the final model behaves with changing height (S7 Fig, S8 Fig). We hope this will help intuition about how the model operates and how the proposed mechanisms produce the observed relationships between height and behavioral measures.

Role of OMR: While their data do not negate a role for the OMR in at least contributing to the “stabilizing” of position relative to the ground (including a forward navigational component), their findings do suggest some additional complexity within the locomotor control system which presumably ensues from some ecological, adaptive needs. I do have trouble translating between the modeling terms/verbiage and the facts on the ground (in the stream actually) in terms of real-world salience. The focus of the paper is “exclusively on…translational OMR”, which is fine, but that does not preclude exploratory, postural and other needs from impacting swim bouts and therefore some of the extracted parameters (perhaps) which might further influence their modeling basis/strategy. Possibly the salience and details of their findings can be better related to the natural OMR, a neural control system of immediate relevance to all moving/sighted vertebrates!

We agree that investigating the OMR in isolation in an impoverished experimental setting where cues other than optic flow have been minimized cannot reveal the full complexity of the OMR, its relation to other behaviors and goals, the role of stimuli other than optic flow, or its full function in the natural setting. (This is addressed in the revised Discussion section “function of the OMR” 815-856). It is however directly relevant to understanding this behavior in important experimental settings such as head-fixed “virtual reality” assays which are even further removed from the natural situation. We also believe that an approach combining experimentation, simulation and modelling with a tight focus on a single aspect can throw light on individual mechanisms (at both algorithmic and neurobiological levels) that in more complex natural situations act together, and that this approach is complementary to a more ethological one.

Misc. Items:

p. 2 where they say “we unpick the detailed relationship”, perhaps the word “unpack” is intended?

Now rephrased with the word “explore”.

Fig. 1C: I was using the vertical dotted lines to look for alignment between parameters. The first dotted line intersects a point of maximal rate of position change (steepest slope) on an upper graph but a point of zero speed on the lower graph. I don’t understand this.

This is an artefact of smoothing applied to the speed trace for presentation purposes only, which introduced a delay in the speed trace. The smoothing also made the profiles of individual bouts appear much too extended and less peaky than they actually are – compare the relative speed profiles which were averaged over many bouts without temporal smoothing. As the speed trace is misleading, we have removed it in favor of showing the raw position data in more detail in what is now Fig 2C and D.

p. 8 (and Fig. 2) Inter-Bout Interval is summarized for all swim bouts analyzed, by pooling across conditions (I think) but in Severi-2018 it appears that IBI varies with whole-field motion speed (unless I am misreading something). I am unsure how this possible issue might impact their modeling and interpretations, but changing IBI would modify average-rate of speed across the channel, a detail not captured by the intensity parameter.

IBI certainly varies with whole-field motion speed, and this is indeed not captured by bout intensity or speed. It is however captured by bout rate: mean bout rate is the reciprocal of the mean interval between one bout and the next – i.e. the sum of active bout duration and interbout interval – and thus reflects variability in the IBI. Part of our argument is that each swim bout has a definite start time, peaks, and then decays gradually to zero rather than having an explicit termination time (like striking a bell, the sound just dies away). As the delineation between the period of active swimming and the interbout interval measure is then somewhat arbitrary we report bout rate instead.

Stripes. I am unsure if they tested different stripe sizes to see if that affects the height-OMR regulation relationship.

We first tested a range of stripe sizes at the intermediate height to find the spatial frequency most effective in eliciting the OMR – 0.015 cycles per degree (cpd), which we used in all subsequent experiments (Methods 900-903, new S9 Fig A). We later compared this frequency with 0.01 and 0.02 cpd at the low and high heights and found that 0.015 cpd was the most effective also at both those heights (906-910, 1262-1274, new S9 Fig B).

Other Food for Thought: The findings of the authors broach a number of interesting topics, which do not particularly need to be addressed in their article, but are provided here mainly for sharing with the authors.

Backward Flow vs. Baseline. Backward Flow can be experienced because swimming is too vigorous, i.e. it is overcompensating for any current flow (so to speak) in which case it should be regulated (slowed) if the goal is to maintain position. But in a low flow environment, backward flow is necessary if the larva is exploring terrain to find food patches. In the modeling it seems like this was addressed by nullifying a single parameter, but in a real stream, it seems these would be two very different goals / intentional behaviors with perhaps distinct control circuits.

Thanks for raising this. In a very low flow environment it may be that the OMR is not elicited at all. This seems likely for the low height low stimulus speed condition in the second experiment, when swimming was much faster than required to stem the stream and faster than that observed for a slightly faster stimulus speed. Perhaps a very low flow allows behaviors other than OMR to emerge which may well have different goals and control circuits. In contrast, when flow is stronger exploration may no longer be so necessary as for example the fish will approach upstream prey as a result of the OMR alone – though a break from OMR will still be needed to engage in final capture (847-849). However, we agree that in a real stream and for at least some range of intermediate flows the OMR will occur alongside other behaviors such as exploration, and that exploring how these interact would be of interest (847-851).

*Baseline Optic Flow* is the visual change when a fish is not swimming (per the bottom two paragraphs on page 6). This descriptions notes that this effect and gain are confounded with height and also that behavior can be studied in a fish that is not swimming, in the course of using an unchanging environment in which height is changed. I know this is very clear in their minds, but as a confounded reader I suspect others might benefit from a bit more context or explanation here.

We have added Fig 1B and further explanation (98-117) which we hope will clarify this.

Height Data, a key discovery of this work, might be considered and expounded upon in a more general context. “Height” is mentioned only three times in the Discussion (all in the first 3 sentences), mainly as a re-statement of the results. Given that the word height appears 76 times in their article, and that the height-effect is their basis for “casting doubt” [p.3] on the idea that the OMR’s main function is to “maintain position”, it would be of value to integrate this height issue into the broader questions about OMR that are addressed in their Discussion.

Height is of direct interest here when reporting the empirical finding that it affects mean swim speeds for a given stimulus speed and thus extent to which the OMR regulates position. Later in the paper the primary focus is to investigate mechanisms underlying the relationship between optic flow and features of the OMR. In that context, height and stimulus speed serve as independent variables we can manipulate to influence optic flow in different ways (both height and stimulus speed impact baseline optic flow while height alone determines the degree to which swimming affects optic flow) rather than being of primary interest in themselves. However, we now do pay more attention again to height specifically in the Discussion section “Function of the OMR” (815-856)

I am curious if some of the deviation away from “perfect” OMR responses seen in the locomotor bouts data reflects a composite function that incorporates a general valuation of “holding station” [or moving upstream into oncoming flow] together with a quite essential need to control posture (by orienting into a perceived flow). Other ecological needs (detecting prey, favorable hunting grounds) might also influence more general navigational computations that incorporate OMR-related signals. The discussion is written as if the different ecological functions of OMR-driven and related behaviors are exclusive as opposed to being contributory and complementary. “Into-stream” swimming is going to mean different things in different circumstances: in a very slow flow regime, larvae can make upstream progress enabling them to explore favorable locations to find food for example. Moderate flow rates might allow position holding, whereas stronger currents / optical flow might preclude this, raising the question of why expend energy in forward swimming if one is moving backwards (relative to the streambed)? Swimming downstream in such cases would greatly accelerate motion through the environment whereas swimming into the current (yielding slower net drift) might in theory allow for better surveillance of local regions where prey might be found. Competing needs and flexible navigational controls should be considered.

While height influences visual perception of the environment and streambed, rheotaxic signals are also being integrated and so a more integrative theory might help to explain their height data and modeling results. Maintaining posture is also crucial, since a larva hit broadside by a current might lose posture and navigational abilities for some bit of time, possibly with significant adverse effects. The OMR swim-channel design is a necessary step to better understanding of this complex and integrated neural control system. But larval zebrafish have multiple and (at times) competing needs including predator avoidance and prey detecting/tracking, ecological factors that must fit into general swim control. Surveillance for prey, minimization of predation risk and assessment of streambed features might all be influenced by height above the streambed; such considerations were not discussed.

Thanks! We agree with all these observations, and now touch on some of them in the Discussion (829-856)

I do not understand how height factors into any of the foregoing considerations nor if the experimental variation of the height parameter (and its modeling) in the OMR-driven swim bouts results in neuronal computations that diverge sharply from more natural sensory processing and sensorimotor integration. An analogous consideration emerges from studies of mammalian visual cortex: natural scenes elicit very different neuronal responses than assorted simple shapes and motions: it is the initial neural responses and processing that will drive further integrative processes.

We agree that extending the model of the translational OMR to include rheotaxis would be a natural next step and the beginning of an attempt to explain how multiple behaviors are orchestrated to meet the concurrent goals you mention. We also agree that height may impact activities other than the OMR, but think that is out of scope for this paper. To explore the extent to which the translational OMR model is sufficient to explain behavior in natural scenes while staying within an experimental/modelling approach, one option as you suggested earlier would be to increase the fidelity of the simulation for example by using stimuli that are images of real stream beds etc to see where the model needs modification to account for behavior in more complex situations.
