# Supplementary material for: A behavioral and modeling study of control algorithms underlying the translational optomotor response in larval zebrafish with implications for neural circuit function
Source: PLoS Comput Biol. 2023 Feb 23;19(2):e1010924. doi: 10.1371/journal.pcbi.1010924 (PMC9998047; doi:10.1371/journal.pcbi.1010924)
Supplement: Attachment — Submitted filename: OMR response.docx [file pcbi.1010924.s014.docx]

Responses to the reviewers

**Reviewer #1**

The authors generally did a good job addressing my concerns. The modeling is clearer now. The description of variables has improved and Figure 4B is very helpful. A few points remain, as listed below.

Major:

Old point 4 on refraction artifacts: I agree with the authors that the results of their additional experiments (Fig. S9) – same spatial frequency peak for all swimming heights – suggests that the issue of stimulus artifacts did not affect the validity of their modeling results. However, to make the experiment reproducible and informative for biologists interested in the psychophysics of this OMR behavior, it would be helpful to be more careful here. The authors argue that the advancing dark edge just below the fish (Kist et al.) is the strongest driver of OMR, which is true. But the existence of a spatial frequency tuning curve shows that the spatial period matters. To address my critique, the authors could calculate how much a single stimulus period (and thereby stimulus speed) at the nadir (just below the fish) gets compressed by refraction artifacts. Using this information, the authors could estimate the relative change in angular speed, which would be a lower but useful estimate of the speed changes (more to the periphery the changes will be larger, but these stimulus positions are less relevant to the fish as the authors point out). This could be done for each different swimming height (i.e. computer-programmed angular speed vs. apparent speed given the artifacts around the nadir). If this change is small, this isn’t much of a problem and could simply be stated in the methods section. If it is larger, the authors should at least mention in the methods section that an offset of the spatial frequency tuning peak (and measured OMR ratios) is likely. The true peak is likely at a position larger than 0.015 cpd. This also means that the OMR ratios in Fig. 2b could be different.

Thank you for alerting us to an error in the previous version which implied that refraction has no effect on perceived spatial frequency for the region immediately below the fish. In fact, as you say, because of refractive effects the peak spatial frequency as perceived by the fish for the stimulus region immediately below is larger than the nominal 0.0156 cpd. For the three height conditions, the perceived spatial frequency at the center of the channel would range from 0.0187 to 0.0210 cpd, (Results 198-200, Methods 921-933, new figure S11 Fig). This was estimated, as you suggest above, by calculating the angle at the eye between rays from the limits of a single stimulus period centered directly below the fish’s eye, while following Dunn & Fitzgerald 2020 in treating the bottom of the channel as an air/water interface. It represents a variation of ±7.7% about the midpoint of the range that was not controlled by increasing the stimulus period in proportion to height. However, the spatial frequency curve (A in S10 Fig) suggests that variations in spatial frequency of up to about 30% around the peak have relatively little effect on the OMR, suggesting that a variation of 7.7% should not have a material effect on the results, and this was confirmed empirically by the subsequent experiment (B in S10 Fig). As argued before, because refractive effects increase towards the periphery this also suggests that it may be the stimulus region below the fish that is most important in determining the strength of the OMR.

Refraction also affects the angular velocity of the image across the retina, and therefore the optic flow experienced during the experiments, although again as you suggest not to a degree that would affect the validity of the modelling. However, it does not impact the OMR ratios reported in Fig. 2 as OMR ratio is defined simply as the ratio between the observed mean swim speed and the linear speed of the stimulus and its calculation does not involve estimation of any other stimulus attributes such as perceived spatial frequency or induced optic flow that are affected by refraction.

New point: Please mention the Github repository containing experimental data, modelling data and program scripts in the paper. The repository will be a great resource for anyone continuing this type of work.

Added at the end of the Methods (1123-1125).

Minor:

Original point 8 on “OMR trajectory” in the context of the over-compensation problem. This comment has been unclear. Let me explain in more detail what I meant: The authors only analyse their so-called “OMR trajectory, which is a ”time series...during which the mean swim speed was at least 0.5 mm/s and the direction of swimming the same as that of the stimulus". So apparently, for the entire of duration of the experiment with motion stimulus, there were periods of zebrafish inactivity which were discarded! Have the authors tried to perform an analysis in which the complete time period during with the moving stimulus was shown is included to calculate the OMR ratio? If the fraction / total amount of time of discarded data is high, then it is possible that the over-compensation problem (for low heights) identified by the authors does on average, over longer time scales, not exist for the fish? Also see line 309: "Bout rate was defined as the frequency of bouts occurring during OMR swim trajectories" So bout rate was only measured during pre-defined "OMR trajectories", not the complete time period.

Our criteria for treating a period of behavior as OMR were deliberately quite strict, as for modelling purposes we wanted to exclude as far as possible periods likely to contain a mixture of behaviors. This is now explained more explicitly in the Results (216-217) and Methods (960-964) sections. During experiment 1, behavior during the test trials qualified as OMR according to those criteria for 73% of the overall time (250). The results of an analysis of the complete time period during trials up to when the fish first reached the end of the channel are now shown in the Results (267-274) and new S2 Fig. The overall pattern is similar to that observed when OMR trajectories only were analyzed, but as expected OMR ratios are generally lower. For the low height condition there is still some suggestion of swimming faster than the stimulus but only at higher stimulus speeds, and averaged over all stimulus speeds the overall OMR ratio was not significantly greater than 1. Thus, as you suggest, when all behaviors are included in the analysis there may not be over-compensation overall when swimming at the low height. Of course, the under-compensation observed when swimming higher up is then even greater, and the conclusion that regulation is only partial and varies systematically with height still holds.

Also, please note that we didn’t mean to imply that over- or under- compensation is in any case necessarily a problem for the fish – we use those terms here merely to denote deviation from the ideal performance of a regulator that tries to match swim speed to stimulus speed. Indeed, a degree of over- or under-compensation during periods of OMR behavior may result in better overall position holding, depending on the context, when those periods occur in alternation with other behaviors. This point is now given more emphasis in the discussion (838-845)

In the following, line numbers correspond to those in the manuscript version with tracked changes.

l.225: value of the used spatial frequency should be indicated in the text.

Added at 195

l.228: refraction had little DIFFERENTIAL effect in the experimental settings, but the tuning peak might be offset altogether (cf. major point 4).

Addressed at 198-200

Fig. 2D, legend: Mention what your definition of OMR ratio is.

Added at 208

l.361 cross-reference Fig S1?

Added at 373

l.510: later -> earlier?

Confusing text now clarified (436) – thank you.

Fig. 6D: add some vertical lines across the 5 sub-panels to ease comparison of events across sub-panels?

Done

**Reviewer #3:**

Review of ZF-OMR Manuscript
Hohman et al. PLoS- Computational Biology -- Resubmission

The authors have made considerable effort to address all points raised in my initial review. I appreciate the extent they went to so as to better convey the nature of the modeling and the height/OMR question. This is evident in new figures (e.g. Figure 4), a more consolidated presentation of the modeling and better discussion of their findings in the context of the natural OMR behavior.

There was some good discussion within their reply concerning the challenges of finding terminology to best describe swim bouts and the vigor of swimming. I do appreciate these challenges but in regards to their now avoiding the term “intensity” in favor of “bout speed” I am confused because the bar graph in the current Figure 5 (if I am looking at the correct figure) has bars for “intensity”, “bout rate” and “swim speed” which adds to my uncertainty.

Sorry for causing confusion – this was just an oversight on our part. “Intensity” has now been replaced with “bout initial speed” in this bar graph Fig 5A and also in Fig 6A in line with the changes elsewhere.

They did incorporate important methodological details on the swim channel and behaviors seen therein. While video of elemental aspects of the behavior would have been nice to include (e.g. within a local portion of the swim channel), we appreciate that kinematics of the OMR behavior have previously been reported.

I remain a bit puzzled about the authors taking an “either/or” approach in their view of the OMR and a goal of disproving the “holding station” hypothesis (lines 816 – 818). I view their nice modeling and data as endorsing a holding-station component, including initial negative movement in relation to “position” when stripes begin moving, and subsequent forward progress which contributes to navigational control of location and posture: two essential behaviors to which the basic OMR response contributes.

We agree that an either/or approach is not appropriate and have made changes to the Abstract (21, 27), Introduction (165-166) and Discussion (826-872) in the hope of making that clear.

In their reply to my reviewer comments there was much good discussion about this topic and different ecological benefits of body and position control. Some of this has been incorporated into their expanded discussion of OMR and related ecological needs and this does make for a stronger work. Overall, their Discussion is fine as is: the authors are entitled to their perspective!
